# Heterologous Combination of VSV-GP and NYVAC Vectors Expressing HIV-1 Trimeric gp145 Env as Vaccination Strategy to Induce Balanced B and T Cell Immune Responses

**DOI:** 10.3389/fimmu.2019.02941

**Published:** 2019-12-18

**Authors:** Beatriz Perdiguero, Carmen Elena Gómez, Juan García-Arriaza, Cristina Sánchez-Corzo, Carlos Óscar S. Sorzano, Sarah Wilmschen, Dorothee von Laer, Benedikt Asbach, Christina Schmalzl, David Peterhoff, Song Ding, Ralf Wagner, Janine Kimpel, Yves Levy, Giuseppe Pantaleo, Mariano Esteban

**Affiliations:** ^1^Department of Molecular and Cellular Biology, Centro Nacional de Biotecnología, Consejo Superior de Investigaciones Científicas, Madrid, Spain; ^2^Biocomputing Unit and Computational Genomics, Centro Nacional de Biotecnología, Consejo Superior de Investigaciones Científicas, Madrid, Spain; ^3^Institute of Virology, Medical University of Innsbruck, Innsbruck, Austria; ^4^Institute of Medical Microbiology and Hygiene, University of Regensburg, Regensburg, Germany; ^5^EuroVacc Foundation, Amsterdam, Netherlands; ^6^Institute of Clinical Microbiology and Hygiene, University Hospital Regensburg, Regensburg, Germany; ^7^Vaccine Research Institute, Créteil, France; ^8^INSERM U955, Paris Est Créteil University, Créteil, France; ^9^AP-HP, Hôpital Henri-Mondor Albert-Chenevier, Service d'Immunologie Clinique et Maladies Infectieuses, Créteil, France; ^10^Division of Immunology and Allergy, Department of Medicine, Centre Hospitalier Universitaire Vaudois and University of Lausanne, Lausanne, Switzerland

**Keywords:** HIV-1 Env, vaccine, VSV-GP, NYVAC, mice immunization, T and B cells, antibodies, immune correlates

## Abstract

The generation of a vaccine against HIV-1 able to induce durable protective immunity continues a major challenge. The modest efficacy (31.2%) of the phase III RV144 clinical trial provided the first demonstration that a prophylactic HIV/AIDS vaccine is achievable but emphasized the need for further refinements of vaccine candidates, formulations, and immunization regimens. Here, we analyzed in mice the immunogenicity profile elicited by different homologous and heterologous prime/boost combinations using the modified rhabdovirus VSV-GP combined with DNA or poxviral NYVAC vectors, all expressing trimeric membrane-bound Env (gp145) of HIV-1 96ZM651 clade C, with or without purified gp140 protein component. In cultured cells infected with recombinant VSV-GP or NYVAC viruses, gp145 epitopes at the plasma membrane were recognized by human HIV-1 broadly neutralizing antibodies (bNAbs). In immunized mice, the heterologous combination of VSV-GP and NYVAC recombinant vectors improved the induction of HIV-1 Env-specific humoral and cellular immune responses compared to homologous prime/boost protocols. Specifically, the combination of VSV-GP in the prime and NYVAC in the boost induced higher HIV-1 Env-specific T cell (CD4/CD8 T cells and T follicular helper -Tfh- cells) immune responses compared to the use of DNA or NYVAC vectors in the prime and VSV-GP in the boost. Such enhanced T cell responses correlated with an enhancement of the Env-specific germinal center (GC) B cell population and with a heavily biased Env-specific response toward the Th1-associated IgG2a and IgG3 subclasses, while the other groups showed a Th2-associated IgG1 bias. In summary, our T and B cell population data demonstrated that VSV-GP-based vectors could be taken into consideration as an optimized immunogenic HIV-1 vaccine candidate component against HIV-1 when used for priming in heterologous combinations with the poxvirus vector NYVAC as a boost.

## Introduction

Since the discovery of the Human Immunodeficiency Virus type 1 (HIV-1) as the causal agent of Acquired Immunodeficiency Syndrome (AIDS) in 1983 (1), many efforts have been performed to discontinue the HIV/AIDS pandemic. The latest estimate indicates that 37.9 million people were living with HIV/AIDS worldwide at the end of 2018 and 770.000 persons died of AIDS in 2018, with sub-Saharan Africa continuing the region most severely affected by the HIV/AIDS pandemic (http://www.unaids.org/en). A significant success in the HIV field has been the development of the life-saving combined antiretroviral therapy (cART) able to suppress the plasma viremia and reduce the risk of HIV-1 transmission (2) and its accessibility to an increasing number of people worldwide. However, the existence of latent HIV-1 reservoirs makes the complete elimination of the virus in cART-treated infected individuals remarkably difficult (3, 4). Therefore, a safe and effective vaccine able to prevent and eliminate the HIV/AIDS pandemic is mandatory but still missing.

To date, only one HIV-1 phase III clinical trial (Thai RV144 trial) has reported some efficacy (31.2%) against HIV-1 acquisition (5). At present, the evaluation of the vaccine-induced immune correlates of protection in non-human primates (NHPs) and in humans, such as broadly neutralizing antibodies (bNAbs), non-neutralizing antibodies targeting the variable loops 1 and 2 (V1/V2) of the HIV-1 envelope (Env), Env-specific polyfunctional antibodies and T cell responses, are guiding different HIV-1 vaccine approaches and regimens (6–8). The RV144 regimen, novel vaccines based on adenovirus vectors, mosaic immunogens, optimized gp140 proteins (such as HIV-1 Env SOSIP trimers) and passive administration of monoclonal antibodies are among the most recent strategies for HIV-1 prevention and treatment.

Since the main objective in the HIV-1 vaccine field is to establish immunization protocols that elicit protective HIV-1-specific responses through the induction of bNAbs together with potent T cell activation, major efforts have been directed to determine the best-in-class combination of both preventive and therapeutic vaccine candidates in preclinical and clinical trials (9–12). The most advanced phase IIb prophylactic clinical studies underway involved the combination of the poxvirus ALVAC vector plus the gp120 protein component (HVTN 702 study; https://www.clinicaltrials.gov/ct2/show/NCT02968849), as well as an adenovirus vector plus the gp140 protein component (HVTN 705/HPX2008 study; https://www.clinicaltrials.gov/ct2/show/NCT03060629). It has to be determined whether these trials will achieve the desirable efficacy.

The recent promising success of ebola epidemic control using a VSV recombinant vector expressing the glycoprotein (GP) of the Zaire strain of ebola virus (13) has elevated the interest in this viral vector. We have recently described a chimeric VSV vector in which the glycoprotein G of VSV has been replaced by the glycoprotein GP of the lymphocytic choriomeningitis virus (LCMV) (VSV-GP) as HIV-1 vaccine vector expressing different forms of HIV-1 Env that induced HIV-1-specific antibodies in mice and rabbits after repeated immunizations with VSV-based recombinant vectors (14). In addition, we have also described in preclinical and clinical trials that prime/boost combinations of a poxvirus vector NYVAC with DNA and Env protein components induced broad HIV-1-specific T and B cell responses (15–18). Both in NHPs and human clinical trials the DNA/NYVAC/protein immunization regimen induced neutralizing antibody responses mainly directed to tier 1 HIV-1 viruses (15–18).

In this work, in an effort to optimize the HIV-1 immunization protocols, we have addressed the question whether VSV-GP and NYVAC vectors expressing trimeric and membrane-bound Env (gp145) protein trigger superior and more balanced HIV-1-specific T and B cell responses in mice when compared with the homologous vector combination, with or without a gp140 protein component, or when DNA was used during priming. Through the analysis of the HIV-1-specific T and B cell populations (CD4/CD8 T cells, T follicular helper -Tfh- and germinal center (GC) B cells and IgG antibody levels and isotypes) in tissues of vaccinated mice, we have established that the combination of VSV-GP/NYVAC is the best-in-class vaccination regimen to achieve a more balanced T and B cell immune responses against HIV-1.

## Materials and Methods

### Cells

Primary chicken embryo fibroblast (CEF) cells, African green monkey kidney cells (BSC-40) and human HeLa cells were grown in Dulbecco's modified Eagle's medium (DMEM) supplemented with 100 μg/ml of streptomycin/100 units/ml of penicillin (Sigma-Aldrich), 0.1 mM non-essential amino acids (Sigma-Aldrich), 2 mM L-glutamine (Merck), 0.5 μg/ml amphotericin B (Fungizone; Gibco-Life Technologies), and 10% heat-inactivated fetal calf serum (FCS; Sigma-Aldrich) for CEF cells or 10% heat-inactivated newborn calf serum (NCS; Sigma-Aldrich) for BSC-40 and HeLa cells. Cells were maintained in a humidified air 5% CO_2_ atmosphere at 37°C.

### Vectors

DNA-based vectors used in this work included the plasmid VRC-8400-gp145(96ZM651) (DNA-gp145; provided by Prof. Dr. Ralf Wagner). The RNA- and codon-optimized 96ZM651 gp145 gene construct was generated using an earlier described gp140 construct (19, 20) encoding a soluble secreted form of the heterotrimeric envelope as template which was then by means of fusion PCR linked to a synthetic DNA fragment encoding the autologous transmembrane domain of the 96ZM651 envelope protein. The resulting PCR product was cloned into the DNA vaccine vector VRC-8400 (PMID: 15994776) via SalI and NotI restriction sites generating the plasmid VRC-8400-gp145(96ZM651). The furin cleavage site was inactivated by a point mutation (REKR to REKS). Plasmids for vaccination were produced in *E. coli* and purified with the EndoFree Plasmid Giga Kit (Qiagen, Hilden, Germany) according to manufacturer's recommendations. The purified plasmids were solubilized in phosphate buffered saline (PBS) at 2 mg/ml and quality controlled regarding identity, supercoil-content, and absence of endotoxin.

VSV-based viruses used in this work included VSV-GP and VSV-GP-gp145 (provided by Dr. Janine Kimpel). VSV-GP has been previously described (21). VSV-GP expressing HIV-1 gp145(96ZM651) protein was constructed by exchanging luciferase gene in VSV-GP-Luc (22) via XhoI/NheI sites with the HIV-1 gp145(96ZM651) cassette obtained by PCR from the above described plasmid VRC-8400-gp145(96ZM651). The resulting virus, VSV-GP-gp145, was recovered via reverse genetics using a helper virus-free protocol. Virus was twice plaque-purified and amplified on Vero cells. Virus supernatants were pelleted through a 20% sucrose cushion via low-speed overnight centrifugation and resuspended in PBS. Virus was stored in aliquots at −80°C and titrated via TCID_50_ assay on BHK-21 cells. For *in vitro* assays, VSV-GP-based viral preparations were retitrated by crystal violet staining plaque assay in BSC-40 cells to calculate the corresponding titers in pfu/ml.

The poxvirus strains used in this work included the genetically attenuated vaccinia virus (VACV)-based vector NYVAC-WT (provided by Sanofi-Pasteur) and the recombinant NYVAC-gp145(96ZM651) expressing a membrane-bound trimeric gp145 from HIV-1 clade C 96ZM651 isolate (NYVAC-gp145). Poxvirus infections were performed with DMEM containing 2% FCS or NCS. Both viruses were grown first in BSC-40 cells and finally in CEF cells and the viral crude preparations obtained were used for the infection of large cultures of CEF cells followed by virus purification through two 36% (w/v) sucrose cushions. Viral titers were calculated by immunostaining plaque assay in BSC-40 cells as previously reported (23) using rabbit polyclonal anti-VACV strain WR antibody (1:1,000; CNB), followed by goat anti-rabbit-horseradish peroxidase (HRP) antibody (1:1,000; Sigma-Aldrich). The viral titer determinations were performed at least 3 times.

### Construction of Plasmid Transfer Vector pLZAW1-gp145(96ZM651)

To generate the plasmid transfer vector pLZAW1-gp145(96ZM651), the corresponding gene from plasmid VRC-8400-gp145(96ZM651) was amplified by PCR introducing PacI and XhoI restriction sites with the primers, and inserting it into pLZAW1. The resulting plasmid pLZAW1-gp145(96ZM651) was kindly provided by Prof. Dr. Ralf Wagner.

### Construction of NYVAC-gp145 Recombinant Virus

The generation of the NYVAC-gp145 recombinant virus was performed by homologous recombination as previously described (19). Briefly, 3 × 10^6^ BSC-40 cells were infected with NYVAC-WT at a multiplicity of infection (m.o.i.) of 0.01 plaque-forming units (pfu)/cell and transfected after 1 h with 6 μg of pLZAW1-gp145(96ZM651) using Lipofectamine-2000 (Invitrogen) according to manufacturer's instructions. After 72 h post-infection (h.p.i.), cells were harvested, lysed by freeze-thaw cycling, sonicated and used for recombinant virus screening along 6 consecutive plaque purification steps in BSC-40 cells. In the first 3 purification steps, NYVAC recombinant viruses containing the HIV-1 gp145 gene and transiently co-expressing the β-galactosidase (β-Gal) marker gene (*lacZ* gene) were selected in the presence of 5-bromo-4-chloro-3-indolyl-β-D-galactopyranoside (X-Gal, 1.2 mg/ml; Sigma-Aldrich). Further propagation of the NYVAC-based recombinant viruses leads to the self-deletion of *lacZ* gene by homologous recombination between the VACV short TK left arm repeat and the TK left arm that are flanking the marker. Therefore, in the last 3 purification steps, NYVAC-based recombinant viruses containing the HIV-1 gp145 gene and having excised the β-Gal marker gene were identified as non-stained viral foci in BSC-40 cells in the presence of X-Gal substrate and isolated.

### PCR Analysis of NYVAC-gp145 Recombinant Virus

To analyze the identity and purity of NYVAC-gp145 viral preparation, DNA was extracted from BSC-40 cells infected with NYVAC-WT or NYVAC-gp145 viruses at 5 pfu/cell for 24 h. Cell membranes were disrupted by proteinase K treatment (0.2 mg/ml proteinase K in 50 mM Tris-HCl pH 8, 100 mM NaCl, 100 mM EDTA pH 8, 1% SDS; 1 h, 55°C), followed by incubation with RNase A (80 μg/ml; PanReac AppliChem). DNA was precipitated using 2-propanol. Primers TK-R: 5′-CTGCCGTATCAAGGACA-3′ and TK-L: 5′-TGATTAGTTTGATGCGATTC-3′ spanning VACV TK flanking regions were used for the PCR analysis of TK locus. The amplification reactions were performed with Phusion High-Fidelity DNA polymerase (BioLabs) according to manufacturer's instructions.

### Analysis of Virus Growth

To assess the virus growth profile, CEF cells grown in 12-well plates were infected with NYVAC-WT or NYVAC-gp145 viruses at 0.01 pfu/cell in duplicate. After virus adsorption, the inoculum was removed and infected cells were incubated with fresh DMEM-2% FCS. At different times post-infection (0, 24, 48, and 72 h), cells were collected by scraping (lysates at 5 × 10^5^ cells/ml), freeze-thawed 3 times and briefly sonicated. Virus titers were calculated by immunostaining plaque assay in BSC-40 cells.

### Fractionation of the HIV-1 gp145 Protein Into Different Virion Compartments After Sequential Detergent Treatment of Purified Recombinant NYVAC Particles

Localization of the HIV-1 gp145 protein in NYVAC-gp145 particles was analyzed by sequential detergent treatment as previously described (24, 25). Briefly, sucrose cushion-purified NYVAC-gp145 virions were solubilized in 0.1 mL of Tris buffer-NP-40 (50 mM Tris-HCl pH 8.5, 10 mM MgCl_2_, 1% non-ionic detergent NP-40). The fraction E1 (soluble lipid envelopes) was harvested by centrifugation, and the remaining pellet was solubilized in 0.1 mL of Tris-buffer-NP-40 plus 50 mM dithiothreitol (DTT). The fraction E2 (soluble protein matrix-like membranes) was collected after centrifugation, and the pellet was solubilized in 0.1 mL of the preceding buffer plus 0.5% deoxycholic acid (DOC) and 0.1% SDS. The fraction E3 (soluble core proteins) was harvested by centrifugation, and the pellet containing the remaining cores (fraction C) was solubilized in 0.1 mL of milliQ H_2_O. The presence of the HIV-1 gp145 protein in the different collected fractions (E1, E2, E3, and C) was determined by western blotting using the rabbit polyclonal anti-gp120 antibody.

### Time-Course Expression of HIV-1 gp145 Protein by Western Blotting Analysis

To analyze the conformation of the HIV-1 gp145 protein expressed by NYVAC-gp145 recombinant virus, monolayers of HeLa cells grown in 24-well plates were infected with NYVAC-WT or NYVAC-gp145(96ZM651) at 5 pfu/cell. At different times post-infection (6 and 24 h), infected cells were harvested, centrifuged at 3,000 rpm for 5 min and resuspended in Laemmli buffer without 2-mercaptoethanol (non-reducing conditions), fractionated by 8% SDS-PAGE and analyzed by western blotting using the rabbit polyclonal anti-gp120 antibody (1:3,000; CNB), followed by goat anti-rabbit-HRP antibody (1:5,000; Sigma-Aldrich) to evaluate the expression of HIV-1 gp145 protein. The immunocomplexes were detected by enhanced chemiluminescence (ECL) system (GE Healthcare).

Next, we compared the expression of HIV-1 gp145 protein by both VSV-GP-gp145 and NYVAC-gp145 vectors in a human cell line. HeLa cells grown in 24-well plates were infected with VSV-GP, VSV-GP-gp145, NYVAC-WT, or NYVAC-gp145 viruses at 5 pfu/cell. At different times post-infection (0, 3, 6, and 16 h), infected cells were harvested and analyzed by western blotting using the rabbit polyclonal anti-gp120 antibody to evaluate the expression of HIV-1 gp145 protein. Poly (ADP-ribose) polymerase (PARP) cleavage and phosphorylation of the eukaryotic translation initiation factor 2α (eIF2α) were also evaluated using the rabbit polyclonal anti-PARP antibody (1:500; Cell Signaling) or the rabbit polyclonal anti-phospho-eIF2α antibody (1:500; Invitrogen), respectively. The rabbit monoclonal anti-β-actin antibody was used as loading control. After incubation with goat anti-rabbit-HRP antibody, the immunocomplexes were detected by ECL system.

### Detection of HIV-1 96ZM651 gp145 Protein on the Surface of Infected Cells

The presence of membrane-bound trimeric HIV-1 gp145 protein on the surface of non-permeabilized infected HeLa cells was determined by flow cytometry using a panel of human bNAbs targeting V1/V2 quaternary N-glycans (PGT145, PG16, PG9, and CH01), V3 N-glycans (10-1074), CD4 binding site (VRC01), gp120/gp41_ECTO_ interface (3BC176), outer domain (OD)-glycans (2G12), or membrane proximal external region (MPER) (10E8) epitopes on the Env trimer. The following non-neutralizing antibodies (non-NAbs) were also included in the analysis: 39F (targeting V3 N-glycans) and F105 (targeting CD4 binding site). Both bNAbs and non-NAbs were obtained through the AIDS Reagent Program, Division of AIDS, NIAID. Monolayers of HeLa cells were infected at 3 pfu/cell with VSV-GP, VSV-GP-gp145, NYVAC-WT, or NYVAC-gp145 viruses. At 16 h.p.i., cells were washed twice with PBS (no calcium/magnesium), detached with PBS 1X−2 mM EDTA, washed with FACS buffer (PBS 1X−2 mM EDTA−1% BSA) and pelleted for 5 min at 1,500 rpm. Cells were then incubated with live/dead fixable red dye (1:200; Invitrogen) for 30 min at 4°C in the dark, washed twice with FACS buffer and blocked with PBS 1X−3% BSA for 30 min at 4°C. 10 μg/ml of each primary human IgG anti-Env bNAb or non-NAb in 50 μl FACS buffer was used to stain 10^6^ cells for 30 min at 4°C in the dark. After incubation, cells were washed twice with FACS buffer and incubated with secondary F(ab′)2-goat anti-human IgG (H+L)-PE antibody (1:100; Beckman Coulter) in 50 μl FACS buffer for 30 min at 4°C in the dark. Cells were then washed twice with FACS buffer and fixed with 0.5% formaldehyde. Samples were acquired in a FC500 Laser flow cytometer (Beckman Coulter) and FlowJo software (Version 10.4.2; Tree Star, Ashland, OR) was used for the analysis of the data. Geometric Mean Fluorescence Intensity (gMFI) values on the “live cells” gate were used to calculate the Env score by applying the formula: No. Env^+^ cells × gMFI/No. live cells.

### Expression of HIV-1 gp145 Protein by Confocal Microscopy Analysis

For the detection of HIV-1 gp145 protein expressed by both VSV-GP-gp145 and NYVAC-gp145 recombinant viruses, monolayers of HeLa cells cultured on glass coverslips at a confluence of 30% were infected with VSV-GP, VSV-GP-gp145, NYVAC-WT, or NYVAC-gp145 at 3 pfu/cell. At 16 h post-infection, cells were washed with PBS and fixed with 4% paraformaldehyde (PFA) in PBS at room temperature (RT) for 15 min. After fixation, coverslips were washed twice with PBS and non-specific unions with antibodies were blocked with PBS 1X−10% FCS for 30 min at RT. After one wash with PBS, human bNAb 10-1074 targeting V3 N-glycans epitope on the Env trimer (10 μg/ml in PBS 1X−10% FCS) was used to stain HIV-1 gp145 protein for 45 min at RT with gentle shaking. Coverslips were then washed 3 times with PBS, blocked for 15 min at RT and incubated with goat anti-human-Alexa488 antibody (green staining; Life Technologies) at a dilution of 1:500 in PBS 1X−10% FCS for 45 min at RT with gentle shaking and in the dark. Cells were washed 3 times with PBS, stained with phalloidin (1:500; Sigma-Aldrich) for 20 min at RT to detect actin, washed with PBS and incubated with 4′,6-diamidino-2-phenylindole (DAPI; 1:200; Sigma-Aldrich) for 20 min at RT to detect cell nuclei. Finally, coverslips were washed with PBS and mounted on glass slides using ProLong Gold anti-fade reagent (Invitrogen). Leica TCS SP5 microscope and the specialized software LasAF (Leica Microsystems) were used for the acquisition of optical sections of the cells and for image recording and processing, respectively.

### Immunogens and Antigens

The protein 97CN54 gp140, manufactured by Polymun (Klosterneuburg, Austria) and provided by Dr. Julie McElrath's group at Fred Hutchinson Cancer Research Center (Seattle, WA, United States), is a purified recombinant clade C envelope protein derived from the clade C/B' strain 97/CN/54 (26). It was administered to mice concurrently with vector immunizations and in the late protein boost mixed up with monophosphoryl lipid A (MPLA) as adjuvant (Polymun; provided by Prof. Dr. Ralf Wagner) and also used to determine the HIV-1 gp140-specific humoral response in immunized mice. The purified protein 96ZM651 gp140, kindly provided by Prof. Dr. Ralf Wagner, was used to determine the HIV-1 gp140-specific cellular (GC and Tfh cells) and humoral responses in immunized mice.

### Preparation of 96ZM651 gp140 Env Trimers

The Env coding sequence from clade C isolate 96ZM651 (GenBank No: AF286224) was codon-optimized for mammalian cell expression (27), the furin cleavage site (REKR) was mutated (REKS) and a C-terminal Flag-His6 tag sequence (SG4SDYKD4KH6) was introduced after position 683 (HXB2 numbering). This gp140 construct was cloned via BsmBI restriction sites into pcDNA3.1_QL expression plasmid (28). FreeStyle™293-F (Life Technologies, Cat. No. R79007) cells were transiently transfected with the expression plasmid by using the Polyethylenimine method (29) (Polysciences, Cat. No. 23966). After 5 days of protein expression, Env was purified from the culture supernatant via a lectin affinity chromatography (Agarose bound *Galanthus nivalis* lectin, Vectorlabs, Cat. No. AL-1243) and subsequent size exclusion chromatography (HiPrep 16/60 Sephacryl S-300 HR, GE Healthcare, Cat. No. GE17-1167-01) operated in PBS. Fractions from size exclusion chromatography were analyzed on gp140 trimer content and the purified trimeric status of the fraction selected was confirmed by Blue Native PAGE (Serva, Cat. No. 43253).

Although the purification procedure of the 97CN54 gp140 protein manufactured by Polymun is not exactly identical, both 96ZM651 and 97CN54 gp140 proteins represent pure trimers, with monomers, dimers and aggregates removed as a result of the respective purification procedures.

### Peptides

The HIV-1 96ZM651 gp140 peptides were obtained through the Centralized Facility for AIDS Reagents, NIBSC, UK. They spanned the HIV-1 gp140 protein from clade C (96ZM651) included in VSV-GP-gp145, NYVAC-gp145, and DNA-gp145 vectors as consecutive 15-mers overlapping by 11 amino acids. They were grouped in three different Env pools: Env-1 (61 peptides), Env-2 (65 peptides), and Env-3 (40 peptides).

### Ethics Statement

Animal experimental protocols were approved by the Ethical Committee of Animal Experimentation (CEEA) of Centro Nacional de Biotecnología (CNB-CSIC, Madrid, Spain) according to Spanish National Royal Decree RD 53/2013, Spanish National Law 32/2007 on animal welfare, exploitation, transport and sacrifice and International EU Guidelines 2010/63/UE on protection of animals used for experimentation and other scientific purposes (permit number PROEX 281/16).

### Mouse Immunization

**Figure 1:**
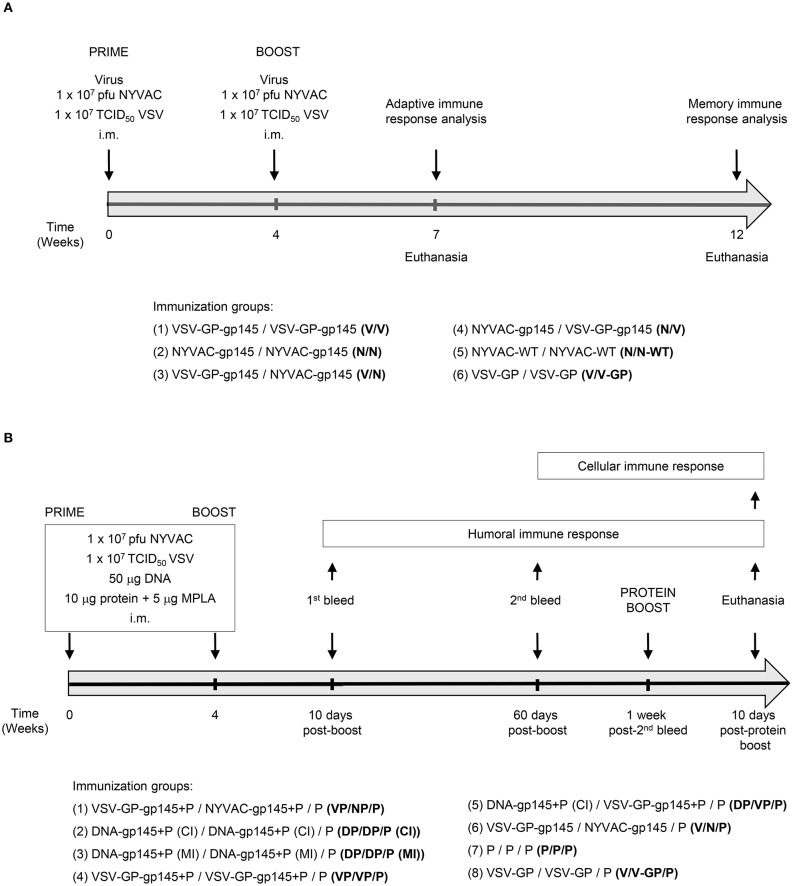
Immunization schedules and groups of mouse studies. **(A)** Mouse Study 1: Groups of female BALB/c mice (*n* = 8) received the indicated doses of VSV-GP-gp145 and NYVAC-gp145 vectors by bilateral i.m. route; 4 weeks later, they received a bilateral i.m. inoculation of the indicated doses of VSV-GP-gp145 and NYVAC-gp145 vectors. At 21 (adaptive) or 56 (memory) days after the last immunization, four animals of each group were sacrificed and spleens were processed to analyze HIV-1 Env-specific T cell responses by ICS assay and sera were harvested to evaluate HIV-1 Env-specific humoral responses by ELISA. **(B)** Mouse Study 2: Groups of female BALB/c mice (*n* = 6) received the indicated doses of VSV-GP and DNA vectors (expressing trimeric HIV-1 clade C gp145 96ZM651 protein) and MPLA-adjuvanted trimeric HIV-1 clade C gp140 97CN54 protein component by i.m. route; 4 weeks later, they received an i.m. inoculation of the indicated doses of VSV-GP-gp145, DNA-gp145, or NYVAC-gp145 vectors and HIV-1 gp140(97CN54) protein. At 10 and 60 days after the last immunization, sera were harvested to analyze HIV-1 gp140-specific humoral responses by ELISA. One week post-second bleed, mice received a HIV-1 gp140(97CN54) protein boost and 10 days post-protein boost, animals were sacrificed and spleens and DLNs were processed to evaluate HIV-1 gp140-specific T and B cell responses by ICS assay and sera were harvested to analyze HIV-1 gp140-specific humoral responses by ELISA.

For the Mouse Study 1, groups of female BALB/c mice (6–8 weeks old; *n* = 8) purchased from ENVIGO were immunized with 1 × 10^7^ pfu of NYVAC-WT or NYVAC-gp145 or 1 × 10^7^ TCID_50_ of VSV-GP or VSV-GP-gp145 viruses by bilateral intramuscular (i.m.) route. Four weeks later, animals were immunized with NYVAC or VSV-GP constructions as in the priming in homologous (groups 1, 2, 5, and 6) or heterologous (groups 3 and 4) combinations and 21 or 56 days after the last immunization, animals were sacrificed and spleens processed for Intracellular Cytokine Staining (ICS) assay and sera collected for Enzyme-Linked Immunosorbent Assay (ELISA) to determine both cellular and humoral adaptive (*n* = 4 at day 21) and memory (*n* = 4 at day 56) immune responses against HIV-1 gp140 protein, respectively. The immunization schedule and the different immunization groups of the Study 1 are shown in Figure 1A.

For the Mouse Study 2, groups of female BALB/c mice (*n* = 6) were immunized with 1 × 10^7^ pfu of NYVAC-gp145, 1 × 10^7^ TCID_50_ of VSV-GP-gp145 or 50 μg of DNA-gp145 vectors concurrently with 10 μg of gp140(97CN54) protein + 5 μg of MPLA as adjuvant by i.m. route (groups 1–5). Four weeks later, animals were immunized with NYVAC, VSV-GP, or DNA constructions as in the priming in homologous (groups 2, 3, and 4) or heterologous (groups 1 and 5) combinations. Group 6 received VSV-GP-gp145 in the prime and NYVAC-gp145 in the boost without HIV-1 gp140(97CN54) protein component (V/N). Group 7 was immunized only with the HIV-1 gp140(97CN54) protein component both in the prime and in the boost (P/P) and group 8 received VSV-GP in the prime and VSV-GP in the boost without HIV-1 gp140(97CN54) protein component (V/V-GP). At 10 and 60 days post-boost, blood was harvested by submandibular bleeding to determine adaptive and memory humoral HIV-1 gp140-specific immune responses, respectively. One week post-second bleed, animals from all groups (1–8) were boosted with 10 μg of gp140(97CN54) protein + 5 μg of MPLA as adjuvant by i.m. route and 10 days post-protein boost, mice were sacrificed and spleens and draining lymph nodes (DLNs) processed for ICS assay and sera harvested for ELISA to determine both cellular and humoral responses against HIV-1 gp140 protein, respectively. Regarding sites of intramuscular immunization: (i) VSV-GP or NYVAC + protein immunization was performed by contralateral injection (CI) (viral vector in one leg and protein in the opposite leg); (ii) DNA + protein regimen was administered by either CI (DNA in one leg and protein in the opposite leg) or mono-lateral injection (MI) (half dose of DNA and protein in the left leg and half dose of DNA and protein in the right leg; injections of DNA and protein were applied approx. 1.5 cm apart; (iii) protein only group was immunized with half dose of the protein in the left leg and half dose of the protein in the right leg. The immunization schedule and the different immunization groups of the Study 2 are shown in Figure 1B.

### Antibody Measurement by ELISA

Antibody binding to HIV-1 gp140 protein in serum was determined by ELISA as previously reported (30). Briefly, individual or pooled sera from immunized mice were serially diluted (2 or 3 fold) in duplicate and incubated with 0.9 μg/ml recombinant clade C HIV-1 gp140(97CN54) or gp140(96ZM651) purified proteins. Levels of Env-specific total IgG binding antibodies or IgG1, IgG2a, or IgG3 subclasses were established as the last serum dilution that gave 3 times the mean optical density value measured at 450 nm (OD_450_ value) of the control group (end point titer) or as the OD_450_ value at a serum dilution of 1:36,000, respectively.

### Analysis of the Cellular Immune Responses by ICS Assay

#### Analysis of HIV-1 gp140-Specific Germinal Center (GC) B Cell Responses

To analyze the magnitude and phenotype of the HIV-1 gp140-specific B cell responses, 2 × 10^6^ cells from the DLNs were seeded on 96-well plates, centrifuged and incubated with Fixable Viability Stain 520 (FVS 520; BD Biosciences). After blocking Fc receptors using anti-CD16/CD32 antibody (BD Biosciences), cells were incubated with 0.3 μg/10^6^ cells of biotinylated clade C 96ZM651 gp140 protein (Biotin-XX Microscale Protein Labeling Kit; Invitrogen) for 30 min at 4°C in the dark. After washing, cells were incubated with the following fluorochrome-conjugated antibodies for surface markers: CD3-FITC, B220-PE-Cy7, IgD-APC-H7, CD38-PerCP-Cy5.5, IgG1-BV421, GL7-Alexa647, IgM-PE-CF594, and CD19-Alexa700 (all from BD Biosciences).

#### Analysis of HIV-1 gp140-Specific T Follicular Helper (Tfh) Cell Responses

For the analysis of the magnitude and phenotype of the HIV-1 gp140-specific Tfh cell responses, 4 × 10^6^ splenocytes (erythrocyte-depleted) were seeded on 96-well plates and stimulated for 6 h in complete RPMI 1640 medium (2 mM L-glutamine, 100 units/ml of penicillin/100 μg/ml of streptomycin, 10 mM Hepes, and 0.01 mM β-mercaptoethanol) with 10% FCS, 1 μl/ml Golgiplug (BD Biosciences), anti-CD154 (CD40L)-PE (BD Biosciences), and 5 μg/ml of HIV-1 96ZM651 clade C Env-1+Env-2+Env-3 peptide pools + 5 μg/ml of HIV-1 96ZM651 gp140 protein. Non-stimulated samples (RPMI) were used as control. After stimulation, cells were washed, stained for surface markers, permeabilized (Cytofix/Cytoperm kit; BD Biosciences) and stained intracellularly. Dead cells were excluded using the violet LIVE/DEAD stain kit (Invitrogen). For the analysis of Tfh cells, the following fluorochrome-conjugated antibodies were used: CD154-PE, IL-4-Alexa488 and IFN-γ-PE-Cy7 for functional analyses and CD4-Alexa700, CD8-V500, PD1(CD279)-APC-efluor780, CXCR5-PE-CF594, and CD44-PE-Cy5 (SPRD) for phenotypic analyses. All antibodies were from BD Biosciences.

#### Analysis of HIV-1 gp140-Specific CD4 and CD8 T Cell Responses

To analyze the magnitude and phenotype of the HIV-1 gp140-specific CD4 and CD8 T cell responses, 4 × 10^6^ splenocytes (erythrocyte-depleted) were seeded on 96-well plates and stimulated for 6 h in complete RPMI 1640 medium with 10% FCS, 1 μl/ml Golgiplug (BD Biosciences), anti-CD107a-FITC (BD Biosciences) and 5 μg/ml of HIV-1 96ZM651 clade C Env-1+Env-2+Env-3 peptide pools. Non-stimulated samples (RPMI) were used as control. After stimulation, cells were washed, stained for surface markers, permeabilized and stained intracellularly. Dead cells were excluded using the violet LIVE/DEAD stain kit (Invitrogen). For the analysis of CD4 and CD8 T cell immune responses, the following fluorochrome-conjugated antibodies were used: CD107a-FITC, IFN-γ-PE-Cy7, IL-2-APC, and TNF-α-PE for functional analyses and CD3-PE-CF594, CD4-APC-Cy7, CD8-V500, CD127-PerCP-Cy5.5, and CD62L-Alexa700 for phenotypic analyses. All antibodies were from BD Biosciences.

For the analysis of the HIV-1 gp140-specific B and T cell immune responses, cells were acquired in a GALLIOS flow cytometer (Beckman Coulter) and the analysis of the data was performed using FlowJo software (Version 10.4.2). The number of lymphocyte-gated events ranged between 10^5^ and 5 × 10^5^.

### Data Analysis and Statistics

For the analysis of ELISA data, a one way ANOVA test followed by Tukey's honest significant difference criterion was carried out. For the analysis of ICS data, a statistical approach that adjusts the values for the non-stimulated controls (RPMI) and calculates the confidence intervals and *p* values was used (31, 32). Only antigen responses significantly higher than the corresponding RPMI samples are represented. All of the values represented are background-subtracted.

## Results

### *In vitro* Characterization of NYVAC- and VSV-GP-Based Recombinant Viruses Expressing HIV-1 gp145 Protein

#### Purity and Virus Growth of NYVAC-gp145 Recombinant Virus

**Figure 2:**
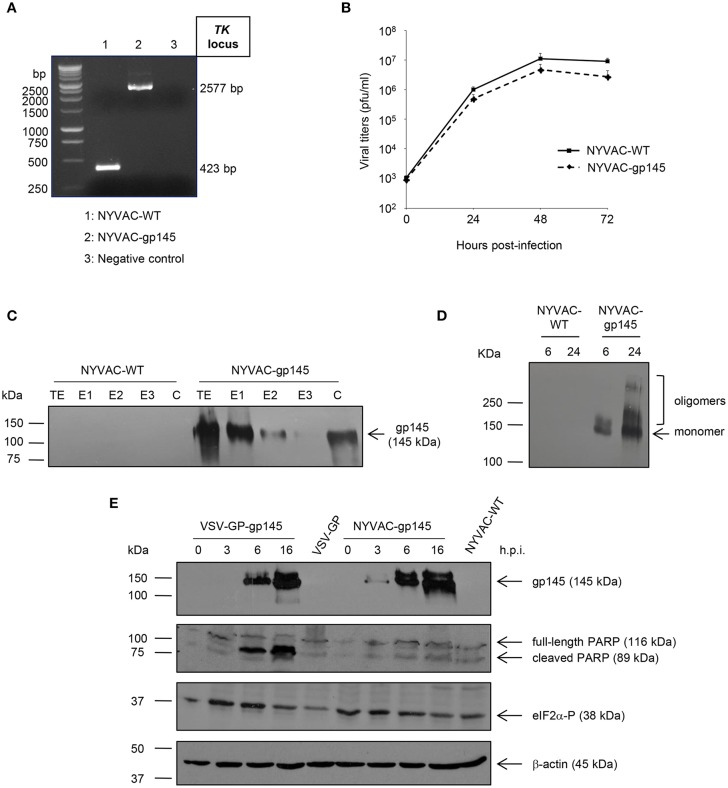
*In vitro* characterization of NYVAC-gp145 and VSV-GP-gp145 recombinant viruses. **(A)** Confirmation of the insertion of the HIV-1 gp145 gene into NYVAC genome by PCR analysis. DNA was extracted from BSC-40 cells infected at 5 pfu/cell with NYVAC-WT or NYVAC-gp145 viruses. Primers TK-L and TK-R spanning VACV *J2R* (TK) flanking regions were used for PCR analysis of TK locus. In parental NYVAC, a 423 bp-product was observed while in NYVAC-gp145 a unique 2577 bp-product was obtained. **(B)** Analysis of the virus growth profile of NYVAC-gp145 virus in a permissive cell line. Primary CEF cells were infected at 0.01 pfu/cell with NYVAC-WT or NYVAC-gp145 viruses. At different times post-infection (0, 24, 48, and 72 h), infected cells were harvested and viral titers were determined by immunostaining plaque assay in BSC-40 cells. Data points represent titers as mean ± SD of *n* = 2 experiments. **(C)** Fractionation of viral proteins and localization of the HIV-1 gp145 protein within purified NYVAC-gp145 particles. Sucrose-purified NYVAC-WT or NYVAC-gp145 virions were disrupted by sequential detergent treatment and different fractions were harvested as described in Materials and Methods. The unfractionated lysate virions (total extract, TE) and the different collected fractions (E1, E2, E3, and C) were analyzed by western blotting using rabbit anti-gp120 antibody. **(D)** Time-course expression of HIV-1 gp145 protein from NYVAC-gp145-infected cells by western blotting analysis. HeLa cells were infected with NYVAC-WT or NYVAC-gp145 viruses at 5 pfu/cell. At different times post-infection (6 and 24 h), cells were harvested, cell extracts fractionated by 8% SDS-PAGE and analyzed by western blotting under non-reducing conditions using the rabbit polyclonal anti-gp120 antibody to assess the expression of HIV-1 gp145 protein. **(E)** Comparative analysis of HIV-1 gp145 protein expression, apoptosis induction, and eIF2α phosphorylation in cells infected with either VSV-GP-gp145 or NYVAC-gp145 viruses by western blotting analysis. HeLa cells were infected with VSV-GP-gp145 or NYVAC-gp145 viruses at 5 pfu/cell. At different times post-infection (0, 3, 6, and 16 h), infected cells were harvested and analyzed by western blotting using the rabbit polyclonal anti-gp120 antibody to assess the expression of HIV-1 gp145 protein, the polyclonal anti-PARP antibody to analyze PARP cleavage or the polyclonal anti-phospho-eIF2α antibody to evaluate the phosphorylation of the transcription factor eIF2α. β-actin was used as loading control.

NYVAC-gp145 recombinant virus was constructed as described under Materials and Methods using NYVAC-WT as parental virus. The correct insertion of HIV-1 gp145(96ZM651) gene into the TK locus of NYVAC genome was confirmed by PCR using primers annealing in TK flanking sequences. As observed in Figure 2A, the HIV-1 gp145(96ZM651) gene was successfully inserted and no wild-type contamination was detected in the NYVAC-gp145 viral preparation.

To determine whether the insertion of HIV-1 gp145(96ZM651) gene into the TK locus of the viral genome affects virus replication, we compared the growth kinetics of the novel NYVAC-gp145 recombinant virus with its parental NYVAC-WT in primary CEF cells. Figure 2B shows that the growth kinetics between parental and recombinant virus were similar, indicating that the expression of HIV-1 gp145 protein does not affect virus growth.

### Incorporation of the HIV-1 gp145 Protein Into Different Compartments Within NYVAC Virions

Since poxvirus preparations used for vaccination mostly consist of intracellular mature virus (MVs), we next wanted to determine whether virus particles isolated from NYVAC-gp145-infected cells incorporated the HIV-1 gp145 protein. To this aim, we analyzed the localization of the HIV-1 Env protein within virion particles after the sequential detergent disruption of purified NYVAC preparations. As it is observed in Figure 2C, the HIV-1 gp145 protein is mainly detected in the E1 (virus membranes) and C (insoluble cores) fractions in NYVAC-gp145 purified virus preparation, highlighting the membrane-bound nature of HIV-1 gp145 protein. The incorporation of similar forms of HIV-1 Env into the VSV-GP virion has been previously reported (14).

### Time-Course Expression of HIV-1 gp145 Protein and Analysis of Apoptosis Induction

To determine the conformation of the HIV-1 gp145 protein expressed by NYVAC-gp145 recombinant virus, a time-course analysis in HeLa cells infected with NYVAC-WT or NYVAC-gp145 viruses under non-reducing conditions was performed by western blotting. As shown in Figure 2D, the 145 kDa monomeric product (HIV-1 gp145 protein) is expressed over time by cells infected with NYVAC-gp145 recombinant virus. Moreover, HIV-1 gp145 protein is also detected with a size compatible with oligomeric forms of the Env protein at late time points (Figure 2D).

Next, we compared the time-course expression of HIV-1 gp145 protein by both VSV-GP-gp145 and NYVAC-gp145 viruses in human HeLa cells infected with VSV-GP, VSV-GP-gp145, NYVAC-WT, or NYVAC-gp145 viruses. Analysis by western blotting confirmed the correct expression of the expected 145 kDa product over time by cells infected with both recombinant viruses (Figure 2E, upper panel). However, gp145 expression is detected earlier (3 h.p.i.) in the extracts from cells infected with NYVAC-gp145 than in those infected with VSV-GP-gp145 where gp145 is detected at 6 h.p.i.

Thereafter, we analyzed the cleavage of PARP to determine the level of apoptosis induced by the infection with VSV-GP or NYVAC recombinant viruses. PARP cleavage generally occurs in apoptotic cells by the activation of caspases. In human PARP, the cleavage separates the PARP's C-terminal catalytic domain (89 kDa) from its N-terminal DNA binding domain (24 kDa) (33). Therefore, this cleavage can be used as an indicator of cells undergoing apoptosis and was analyzed by western blotting in HeLa cells infected with VSV-GP, VSV-GP-gp145, NYVAC-WT, or NYVAC-gp145 viruses (5 pfu/cell) at 0, 3, 6, and 16 h.p.i. using an antibody that recognizes both forms of the protein, cleaved and full-length PARP. As shown in the second panel of Figure 2E, the 116-kDa full-length PARP was mostly cleaved (89 kDa) in cells infected with VSV-GP-gp145 at 6 and 16 h.p.i., while in cells infected with NYVAC-gp145 this cleavage was reduced at this late time-point, indicating that VSV-GP-gp145 induced a higher level of apoptosis than NYVAC-gp145.

In response to cellular stress conditions and apoptosis, such as virus infection, the α subunit of the initiation factor eIF2 can be phosphorylated by a number of related protein kinases (34). Since the phosphorylated form of eIF2α inhibits the guanine nucleotide exchange factor eIF2B and blocks the recycling of eIF2 between successive rounds of protein synthesis, massive phosphorylation of eIF2α can lead to the downregulation of the overall rate of protein synthesis as part of the anti-viral response triggered by the cell in response to infection (34). Therefore, to determine *in vitro* the effect of VSV-GP or NYVAC infection, we analyzed the phosphorylation level of eIF2α in HeLa cells infected with VSV-GP, VSV-GP-gp145, NYVAC-WT, or NYVAC-gp145 viruses (5 pfu/cell) at 0, 3, 6, and 16 h.p.i. As shown in the third panel of Figure 2E, the level of eIF2α phosphorylation is increased at early time post-infection (3 and 6 h.p.i.) in VSV-GP-gp145-infected cells while it remains unaltered in the case of NYVAC-gp145 infection, indicating that VSV-GP infection is more effective in the activation of eIF2α phosphorylation, correlating with apoptosis induction.

### Genetic Stability

The stability of the HIV-1 gp145 protein expressed by NYVAC-gp145 virus was analyzed by serial passage of the recombinant on primary CEF cells, isolating 30 individual viral plaques at passage 13 and confirming by western blotting that all plaques correctly expressed the full-length HIV-1 gp145 protein. Similarly, the stability of the HIV-1 gp145 protein expressed by VSV-GP-gp145 virus was confirmed by serial passage of the recombinant on Vero cells, isolating 10 individual viral plaques at passage 10 and confirming by western blotting and immunostaining that all plaques correctly expressed the full-length HIV-1 gp145 protein (data not shown).

### bNAbs Binding Profile to Membrane-Bound HIV-1 gp145 Protein

**Figure 3:**
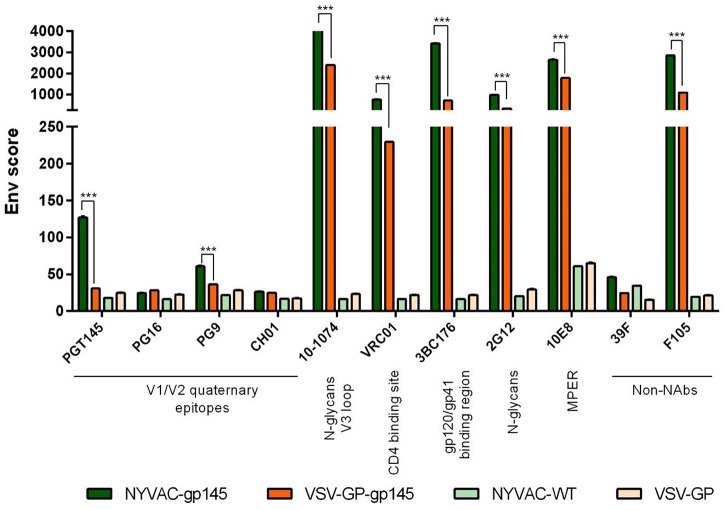
bNAb binding profile to membrane-bound trimeric HIV-1 gp145 protein by flow cytometry. Monolayers of HeLa cells infected with VSV-GP, VSV-GP-gp145, NYVAC-WT, or NYVAC-gp145 viruses at 3 pfu/cell for 16 h were processed for flow cytometry as detailed in Materials and Methods using 10 μg/ml of each primary human IgG anti-Env bNAb and non-NAb. The selected panel of human bNAbs targets V1/V2 quaternary N-glycans (PGT145, PG16, PG9, and CH01), V3 N-glycans (10-1074), CD4 binding site (VRC01), gp120/gp41_ECTO_ interface (3BC176), outer domain (OD)-glycans (2G12), or MPER (10E8) epitopes on the Env trimer. The human non-NAbs included in the analysis targets V3 N-glycans (39F) and CD4 binding site (F105). Samples were acquired in a FC500 Laser flow cytometer and geometric Mean Fluorescence Intensity (gMFI) values on the “live cells” gate were used to analyze the data. The Env score results from applying the formula: No. Env^+^ cells × gMFI/No. live cells. Data from one experiment representative of two performed are shown. ^***^p < 0.001.

To analyze whether the HIV-1 Env protein expressed by both VSV-GP-gp145 and NYVAC-gp145 recombinant viruses exhibits a membrane-bound trimeric conformation, we further evaluated by flow cytometry the affinity of a panel of bNAbs and non-NAbs to the HIV-1 gp145 protein expressed on the plasma membrane of VSV-GP-gp145- or NYVAC-gp145-infected cells. The selected panel of bNAbs targets the majority of the vulnerable HIV-1 Env trimer epitopes reported (35). As observed in Figure 3, all the bNAbs assayed, except PG16 and CH01, specifically recognized the HIV-1 gp145 protein expressed by NYVAC-gp145 virus, while in the case of VSV-GP-gp145, none of the bNAb targeting V1/V2 quaternary epitopes recognized the Env protein expressed from VSV-GP backbone. For the rest of the bNAbs assayed, different affinities were observed with higher Env scores in the case of NYVAC-gp145-infected cells compared with VSV-GP-gp145-infected cells. For NYVAC-gp145-infected cells, bNAbs targeting V3 N-glycans (10-1074), CD4 binding site (VRC01), gp120-gp41 interface (3BC176), N-glycans (2G12), and MPER region (10E8) epitopes displayed higher Env scores than bNAbs recognizing V1/V2 quaternary epitopes (PGT145 and PG9). Regarding the recognition of HIV-1 gp145 protein by non-NAbs, F105 recognizes both HIV-1 gp145 proteins while 39F does not. Overall, these results demonstrated that the HIV-1 gp145 protein expressed by either VSV-GP-gp145 or NYVAC-gp145 viruses was inserted into the plasma membrane of infected cells in a conformation with a favored exposure of bNAb epitopes.

### Membrane Localization of HIV-1 gp145 Protein by Confocal Microscopy

**Figure 4:**
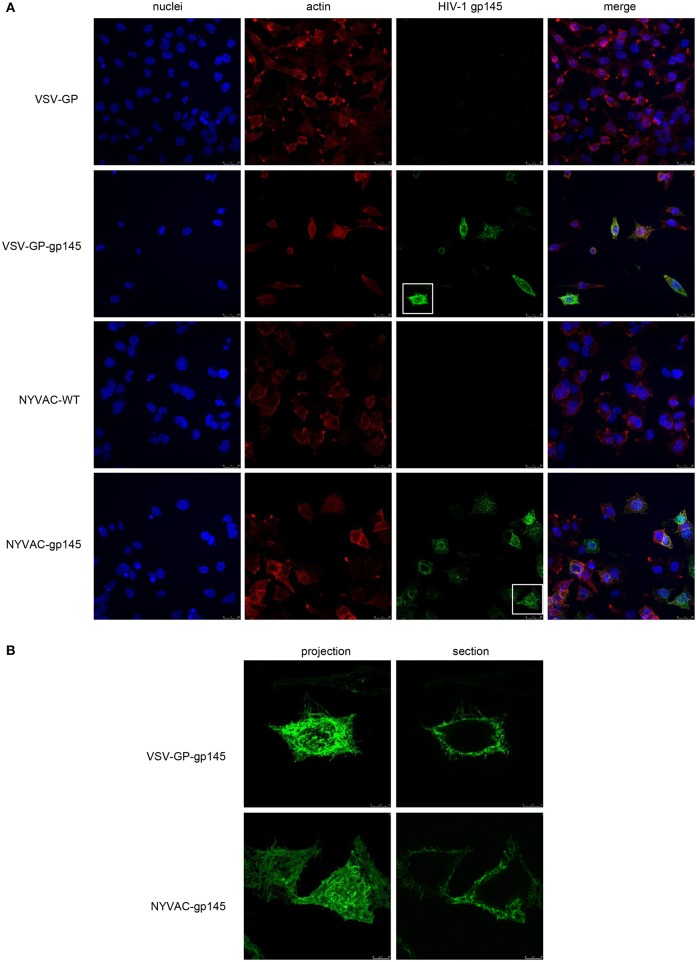
Membrane localization of HIV-1 gp145 protein by confocal microscopy. **(A)** HeLa cells were infected at 3 pfu/cell with VSV-GP, VSV-GP-gp145, NYVAC-WT, or NYVAC-gp145 viruses. At 16 h.p.i., cells were fixed and incubated with human 10-1074 bNAb. After incubation with the corresponding secondary antibody conjugated with Alexa Fluor 488 (green), cells were stained with phalloidin and DAPI to detect F-actin (red) and cell nuclei (blue), respectively, and visualized by confocal microscopy. Scale bar: 25 μm. **(B)** Membrane pattern of HIV-1 gp145 protein in VSV-GP-gp145- (upper) or NYVAC-gp145- (lower) infected cells. The images show a magnification of the single cells indicated in section **(A)** as projections (left) or cross-sections (right). Scale bar: 8 μm.

The expression and membrane localization of HIV-1 gp145 protein expressed by either VSV-GP-gp145 or NYVAC-gp145 recombinant viruses was determined by immunofluorescence analysis in non-permeabilized HeLa cells infected for 16 h with the different parental and recombinant viruses and incubated with the human bNAb 10-1074 targeting V3 N-glycans epitope on the Env trimer, as well as with specific markers for cell nuclei (DAPI) and actin (phalloidin). As observed in Figure 4, the HIV-1 gp145 protein (in green) expressed by VSV-GP-gp145- or NYVAC-gp145-infected cells is specifically recognized by 10-1074 bNAb (Figure 4A, third column) showing a gross punctate pattern in projection or cross-section images of single Env^+^ cells (Figure 4B). This result confirms the membrane-bound trimeric nature of the HIV-1 gp145 protein.

### HIV-1 Env-Specific Immune Response Induced by Homologous or Heterologous Prime/Boost Immunizations With VSV-GP-gp145 and NYVAC-gp145 Vectors

To assay *in vivo* the immunogenicity against HIV-1 gp145(96ZM651) protein elicited by the immunization with different combinations of VSV-GP-gp145 and NYVAC-gp145 recombinant vectors, we determined the HIV-1 Env-specific responses induced in mice after a prime/boost immunization protocol. BALB/c mice, 8 in each group, were immunized with 1 × 10^7^ pfu of NYVAC-WT or NYVAC-gp145, or 1 × 10^7^ TCID_50_ of VSV-GP or VSV-GP-gp145 viruses by i.m. route. Four weeks later, mice were immunized with NYVAC or VSV-GP constructs as in the priming in homologous (groups 1, 2, 5, and 6) or heterologous (groups 3 and 4) combinations and 21 (adaptive phase) or 56 (memory phase) days post-boost, mice were sacrificed (*n* = 4 at each time-point) and spleens processed for ICS assay and sera harvested for ELISA to measure the HIV-1 Env-specific cellular and humoral responses, respectively (Figure 1A).

#### HIV-1 Env-Specific Humoral Immune Response Elicited by Homologous or Heterologous Combinations of VSV-GP-gp145 and NYVAC-gp145 Vectors

The HIV-1 gp145(96ZM651) protein expressed by VSV-GP-gp145 and NYVAC-gp145 vectors has been optimized to be delivered as membrane-bound Env trimers. Therefore, we decided to characterize the HIV-1 Env-specific humoral response elicited by different combinations of VSV-GP and NYVAC recombinant vectors expressing this optimized HIV-1 Env antigen. The reactivity of serum from individual mice against purified trimeric HIV-1 gp140(96ZM651) protein at the adaptive and memory phases was quantified by ELISA.

**Figure 5:**
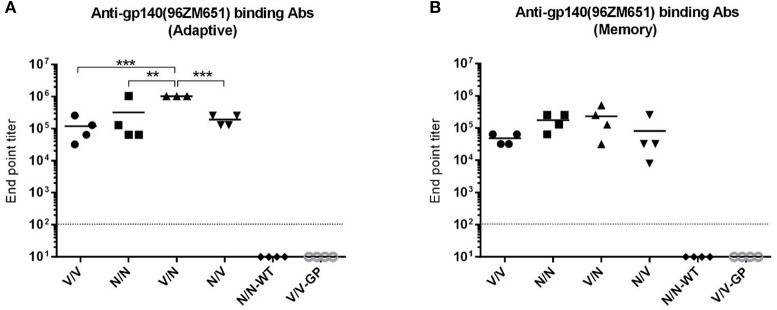
Adaptive and memory HIV-1 gp140-specific humoral responses induced after different homologous or heterologous prime/boost immunizations of mice with VSV-GP and NYVAC vectors expressing trimeric HIV-1 gp145(96ZM651) protein. Levels of HIV-1 gp140-specific total IgG binding antibodies measured in serum from immunized animals at 21 **(A)** or 56 **(B)** days post-boost by ELISA. The different shapes represent the antibody titers for each individual mice defined as the last serum dilution that gave three times the mean OD_450_ value of the control group (end point titer), the solid line indicates the mean antibody titer for each group and the dotted line represents the detection limit of the assay. One mouse was lost in the group (V/N) at 21 days. ^**^p < 0.005; ^***^p < 0.001.

At 21 days post-boost (Figure 5A), serum from all mice receiving VSV-GP and NYVAC vectors expressing the HIV-1 gp145 protein (groups 1 to 4) exhibited high titers of HIV-1 gp140-specific total IgG binding antibodies with magnitudes ranging from 10^4^ to 10^6^ whereas VSV-GP or NYVAC-WT control groups showed no reactivity (*p* < 0.001), indicating the immunogenic nature of both vectors. Moreover, mice immunized with the heterologous combination VSV-GP-gp145/NYVAC-gp145 elicited the highest level of HIV-1 gp140-specific total IgG binding antibodies, with the differences being statistically significant. This result was also observed during the memory phase although the differences were not significant (Figure 5B). Furthermore, the levels of HIV-1 gp140-specific total IgG binding antibodies in the different immunization groups declined (2–4 fold) with time compared to the levels detected in the adaptive phase (Figures 5A,B).

#### HIV-1 Env-Specific CD4 and CD8 T Cell Immune Responses Elicited by Homologous or Heterologous Combinations of VSV-GP-gp145 and NYVAC-gp145 Vectors

Since T cell immune responses also play a key role in the control of HIV-1 infection, we next evaluated the HIV-1 gp140-specific CD4 and CD8 T cell responses induced by VSV-GP or NYVAC recombinant vectors expressing the HIV-1 gp145(96ZM651) protein. Splenocytes from immunized animals were stimulated *ex vivo* for 6 h with HIV-1 gp140(96ZM651) Env-1+Env-2+Env-3 peptide pools and then cells were incubated with specific antibodies to identify T cell lineage (CD3, CD4, and CD8), antigen-specific cytokine response (IL-2, IFN-γ, and TNF) and degranulation (CD107a). The percentages of T cells with CD4 or CD8 phenotype that produced IL-2 and/or IFN-γ and/or TNF and/or expressed CD107a defined the overall HIV-1 gp140-specific CD4 or CD8 T cell immune responses.

**Figure 6:**
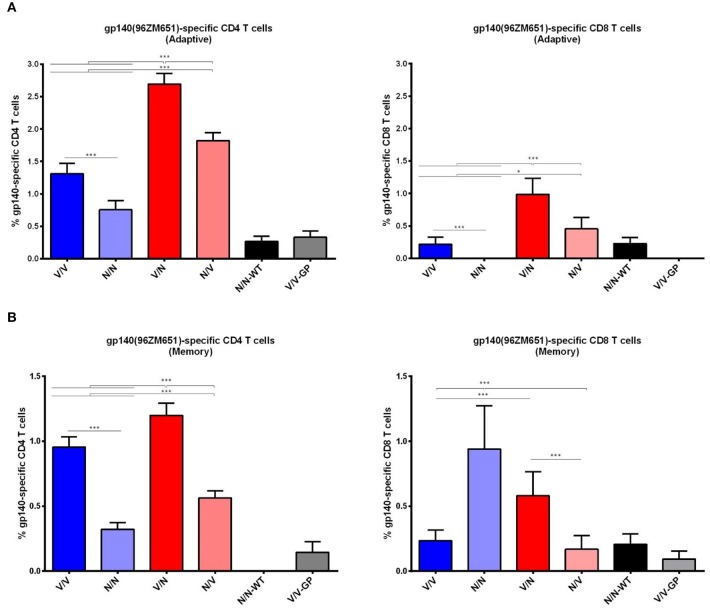
Adaptive and memory HIV-1 gp140-specific CD4 and CD8 T cell responses induced after different homologous or heterologous prime/boost immunizations of mice with VSV-GP-gp145 and NYVAC-gp145 vectors. Magnitude of the HIV-1 gp140-specific CD4 (left) or CD8 (right) T cell responses measured at 21 **(A)** or 56 **(B)** days post-boost by ICS assay following stimulation of splenocytes derived from immunized mice with HIV-1 gp140(96ZM651) peptide pools Env-1+Env-2+Env-3. The total value in each group represents the sum of the percentages of CD4^+^ or CD8^+^ T cells secreting IL-2 and/or IFN-γ and/or TNF and/or expressing CD107a against HIV-1 gp140(96ZM651) peptide pools. All data are background subtracted. 95% Confidence Interval (CI) is represented. ^*^p < 0.05; ^***^p < 0.001.

The HIV-1 gp140-specific T cell responses in the adaptive phase were polarized toward the CD4 compartment in all immunization groups (Figure 6A). In mice immunized with the heterologous VSV-GP-gp145/NYVAC-gp145 or NYVAC-gp145/VSV-GP-gp145 combinations (depicted in red), CD4^+^ and CD8^+^ T cell immune responses were higher than those elicited in animals immunized with the homologous VSV-GP-gp145 or NYVAC-gp145 combinations (depicted in blue) (*p* < 0.001). Moreover, the administration of VSV-GP-gp145 in the prime and NYVAC-gp145 in the boost induced the highest percentage of HIV-1 gp140-specific CD4 and CD8 T cells (*p* < 0.001). Regarding homologous combination, VSV-GP-gp145/VSV-GP-gp145 regimen induced higher CD4^+^ or CD8^+^ T cells compared to the homologous repeated administration of NYVAC-gp145 vector (*p* < 0.001) with CD8^+^ T cells being undetectable in the group immunized with the homologous NYVAC-gp145 regimen (Figure 6A, right panel). In the memory phase, HIV-1 gp140-specific T cell responses were polarized toward the CD4 compartment in all immunization groups except in homologous NYVAC-gp145/NYVAC-gp145 regimen in which this response was polarized toward the CD8 subset (Figure 6B). For CD4 T cells, the heterologous combination VSV-GP-gp145/NYVAC-gp145 elicited again the highest HIV-1 gp140-specific response (*p* < 0.001) while for CD8 T cells the homologous NYVAC-gp145/NYVAC-gp145 and the heterologous VSV-GP-gp145/NYVAC-gp145 immunizations induced the highest HIV-1 gp140-specific responses (*p* < 0.001).

In addition, we also evaluated the phenotype of the HIV-1 gp140-specific T cell responses by analyzing the expression of CD127 and CD62L surface markers, which allows the identification of the following T cell memory subpopulations: T effector (TE; CD127^−^CD62L^−^), T effector memory (TEM; CD127^+^CD62L^−^) and T central memory (TCM; CD127^+^CD62L^+^). During adaptive and memory phases, the HIV-1 gp140-specific CD4 and CD8 T cell responses were mostly of TEM phenotype in all immunization groups (data not shown).

Overall, these results showed that the heterologous combination of VSV-GP-gp145 in the prime followed by NYVAC-gp145 in the boost is the best immunization regimen to elicit HIV-1 Env-specific humoral and CD4/CD8 T cell responses both during adaptive and memory phases of the immune response.

#### Comparison of the HIV-1 Env-Specific Immune Response Induced by Different Homologous or Heterologous Prime/Boost Immunization Protocols Using VSV-GP-gp145 in Combination With DNA-gp145 or NYVAC-gp145 Vectors, With or Without a gp140 Protein Component

Once the combination of VSV-GP-gp145 in the prime and NYVAC-gp145 in the boost was established as the optimal immunization regimen for the induction of high levels of HIV-1 Env-specific humoral and cellular responses, we decided to determine the effect of including an adjuvanted gp140 protein component on the enhancement of the humoral response, as well as to test the impact of a DNA vector expressing the same optimized HIV-1 gp145 protein on the HIV-1 gp140-specific cellular response.

For this purpose, groups of BALB/c mice (*n* = 6) were immunized with 1 × 10^7^ pfu of NYVAC-gp145, 1 × 10^7^ TCID_50_ of VSV-GP-gp145 or 50 μg of DNA-gp145 vectors concurrently with 10 μg of HIV-1 gp140(97CN54) protein + 5 μg of MPLA as adjuvant by i.m. route (groups 1–5). Four weeks later, mice were immunized with NYVAC-gp145, VSV-GP-gp145, or DNA-gp145 constructs as in the priming in homologous (groups 2, 3, and 4) or heterologous (groups 1 and 5) combinations. Group 6 received VSV-GP-gp145 in the prime and NYVAC-gp145 in the boost without the gp140(97CN54) protein component (V/N). Group 7 was immunized only with the gp140(97CN54) protein component both in the prime and in the boost (P/P) and group 8 received empty VSV-GP in the prime and in the boost without the gp140(97CN54) protein component (V/V-GP). At 10 and 60 days post-boost, the HIV-1 gp140-specific antibody response was analyzed. One week after the second bleed, animals from all groups (groups 1–8) were boosted with 10 μg of HIV-1 gp140(97CN54) protein + 5 μg of MPLA by i.m. route. Ten days post-protein boost, animals were sacrificed, spleens and DLNs processed for ICS assay and sera harvested for ELISA to measure cellular (GC B cells, Tfh cells and CD4/CD8 T cells) and humoral responses against HIV-1 gp140 protein, respectively (Figure 1B).

#### HIV-1 Env-Specific Humoral Immune Response

Based on this new modified prime/boost immunization schedule with DNA-gp145 and HIV-1 gp140(97CN54) protein components, we decided to analyze the humoral response induced by the different combinations of VSV-GP-gp145, NYVAC-gp145, and DNA-gp145 vectors against HIV-1 gp145(96ZM651) protein, expressed by the vectors, or HIV-1 gp140(97CN54) protein administered concurrently with VSV-GP-, NYVAC-, and DNA-based vectors and in the late protein boost adjuvanted with MPLA. The reactivity of serum at 10 days post-boost (d10), 60 days post-boost (d60), or 10 days post-protein boost (d10 post-protein boost) against purified trimeric HIV-1 gp140(96ZM651) or gp140(97CN54) proteins was quantified by ELISA.

**Figure 7:**
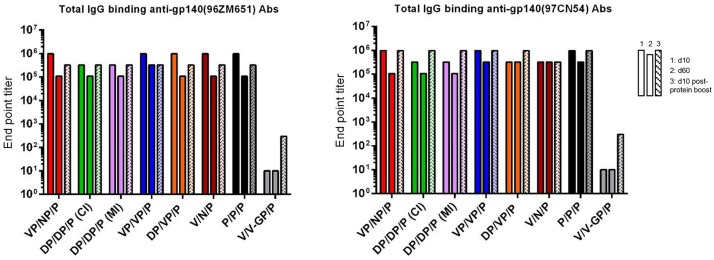
Adaptive and memory HIV-1 gp140-specific humoral responses induced after different homologous or heterologous prime/boost immunizations of mice with VSV-GP-gp145 in combination with DNA-gp145 or NYVAC-gp145 vectors, co-administered with a gp140(97CN54) protein component. Levels of HIV-1 gp140(96ZM651)-**(left)** or gp140(97CN54)-**(right)** specific total IgG binding antibodies measured in pooled sera from immunized mice at 10 (first column) or 60 (second column) days post-boost or 10 days post-protein boost (third column) by ELISA.

**Figure 8:**
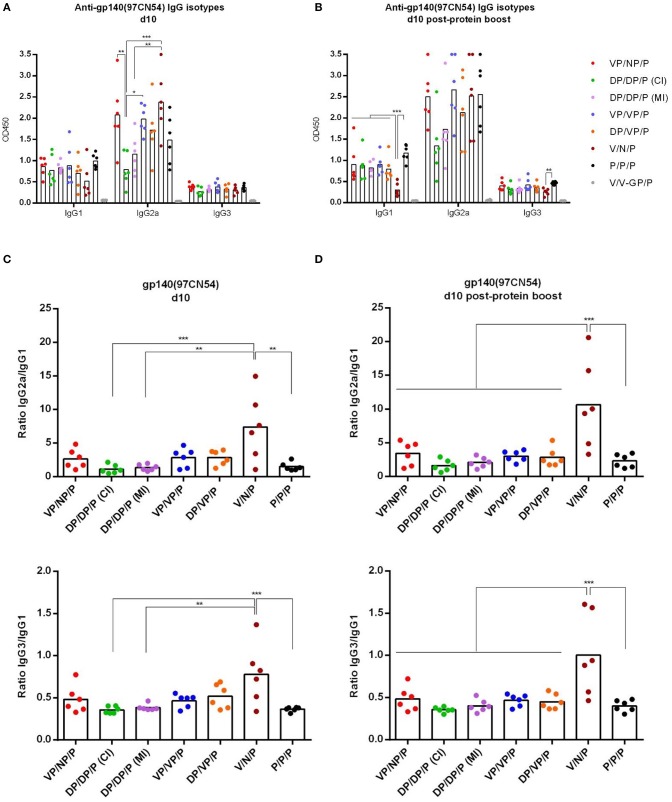
Analysis of the HIV-1 gp140-specific humoral responses induced after prime/boost immunizations of mice with VSV-GP-gp145 in combination with DNA-gp145 or NYVAC-gp145 vectors, co-administered with a gp140(97CN54) protein component. Levels of HIV-1 gp140(97CN54)-specific IgG1, IgG2a, and IgG3 binding antibodies measured in serum from immunized individual mice at 10 days post-boost (d10) **(A)** or 10 days post-protein boost (d10 post-protein boost) **(B)** measured as OD_450_ at a serum dilution of 1:36,000 by ELISA. **(C,D)** Ratios IgG2a/IgG1 (upper) or IgG3/IgG1 (lower) at d10 **(C)** or d10 post-protein boost **(D)**. ^*^p <0.05; ^**^p <0.005; ^***^p <0.001.

As shown in Figure 7, at d10 all immunization regimens induced high levels of HIV-1 Env-specific total IgG binding antibodies against both Env proteins with titers ranging from 10^5^ to 10^6^, decreasing by about 0.5–1 log at d60 and being boosted by the late gp140 protein immunization. Next, we performed a more detailed analysis of the humoral response induced by the different immunization groups at d10 and d10 post-protein boost against the HIV-1 gp140(97CN54) protein (Figure 8). First, we determined the IgG subclasses (IgG1, IgG2a, and IgG3) induced by the different vector combinations. As shown in Figures 8A,B, all immunization regimens induced HIV-1 gp140(97CN54)-specific humoral responses mainly mediated by the IgG2a subclass at d10 and d10 post-protein boost. At 10 days post-boost (Figure 8A), the levels of HIV-1 Env-specific IgG2a binding antibodies were significantly higher in VSV-GP-gp145-primed groups than those observed in DNA-gp145-primed groups. This result was also observed at d10 post-protein boost (Figure 8B), although the differences between VSV-GP-gp145- and DNA-gp145-primed groups were not statistically significant. After protein boost, the levels of HIV-1 gp140(97CN54)-specific IgG1 antibodies observed in the group VSV-GP-gp145/NYVAC-gp145 without the gp140 protein component (V/N/P) were lower compared with the values obtained in the rest of the groups (Figure 8B).

Finally, we analyzed IgG2a/IgG1 or IgG3/IgG1 ratios in the different groups at d10 (Figure 8C) or d10 post-protein boost (Figure 8D). We observed that the group VSV-GP-gp145/NYVAC-gp145 without the gp140 protein component (V/N/P) heavily biased the HIV-1 Env-specific response toward the Th1-associated IgG2a and IgG3 subclasses while the other groups showed a Th2-associated IgG1 bias.

#### HIV-1 Env-Specific GC B Cell Immune Response

Germinal centers (GCs) are described as secondary lymphoid structures within B cell follicles where B cells undergo affinity maturation and class-switch recombination to produce high-affinity antibodies (36–39). Therefore, we next characterized by flow cytometry the GC B cell population induced by the different immunization regimens in DLNs at 10 days post-protein boost. The gating strategy used for the identification of GC B cells was the following: after gating on singlets/lymphocytes/live/CD3^−^CD19^+^ cells, B cells were identified as B220^+^ cells and on these we defined GC B cells as GL7^+^CD38^−^ population. Biotinylated HIV-1 gp140(96ZM651) protein was used to identify the frequency of HIV-1 Env-specific GC B cells (Supplementary Figure 1A).

**Figure 9:**
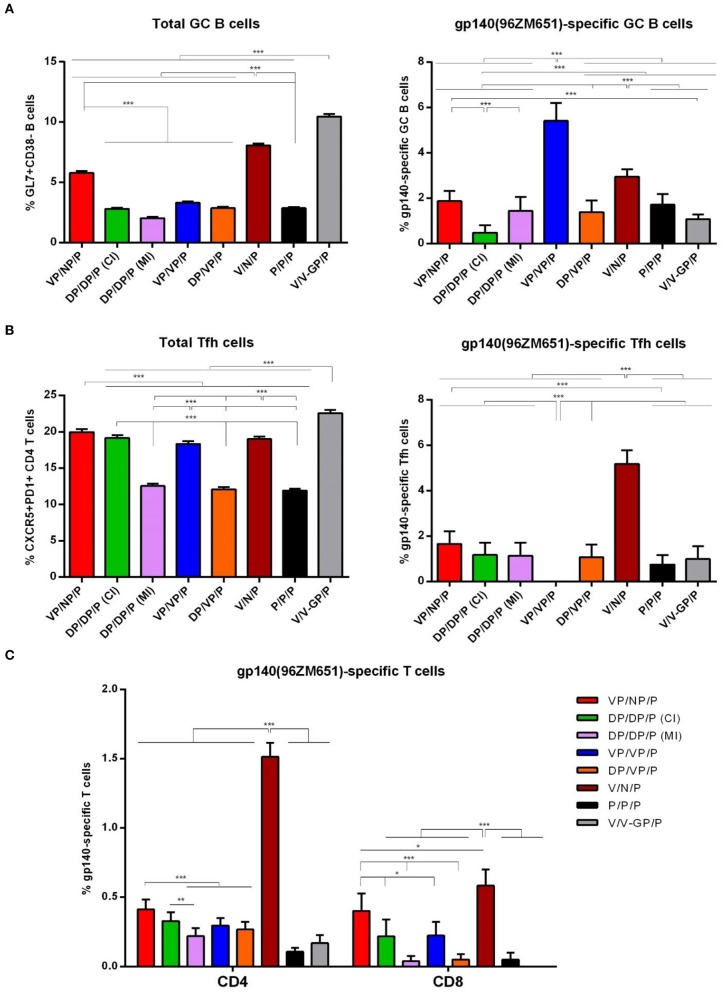
HIV-1 gp140-specific B and T cell responses induced after prime/boost immunization of mice with VSV-GP-gp145 in combination with DNA-gp145 or NYVAC-gp145 vectors, co-administered with a gp140(97CN54) protein component. **(A)** Left: Magnitude of the total GC B cells (GL7^+^CD38^−^) in DLNs measured 10 days post-protein boost by ICS assay following incubation of lymphocytes derived from immunized animals with biotinylated HIV-1 gp140(96ZM651) protein. Right: Magnitude of the HIV-1 gp140-specific GC B cells in DLNs measured as in left. **(B)** Left: Magnitude of the total CD4 T cells with Tfh phenotype (CXCR5^+^PD1^+^) in spleen measured 10 days post-protein boost by ICS assay in non-stimulated (RPMI) splenocytes derived from immunized mice. Right: Magnitude of the HIV-1 gp140-specific Tfh cells in spleen measured 10 days post-protein boost following stimulation of splenocytes with HIV-1 gp140(96ZM651) peptide pools + HIV-1 gp140(96ZM651) protein. The total value in each group represents the sum of the percentages of Tfh^+^ cells secreting IL-4 and/or IFN-γ and/or expressing CD40L against both HIV-1 gp140(96ZM651) peptide pools and protein. Data in right are background subtracted. **(C)** Magnitude of the HIV-1 gp140-specific CD4 or CD8 T cell responses measured 10 days post-protein boost by ICS assay following stimulation of splenocytes derived from immunized mice with HIV-1 gp140(96ZM651) peptide pools. The total value in each group represents the sum of the percentages of CD4^+^ or CD8^+^ T cells secreting IL-2 and/or IFN-γ and/or TNF and/or expressing CD107a against HIV-1 gp140(96ZM651) peptide pools. All data are background subtracted. 95% CI is represented ^*^p < 0.05; ^**^p < 0.005; ^***^p < 0.001.

As shown in Figure 9A (left panel), the i.m. administration of the different combinations of VSV-GP-gp145, DNA-gp145, and NYVAC.gp145 vectors induced high levels of total GC B cells in DLNs compared with the 0.4% of total GC B cells detected in naïve mice, although some differences were detected between groups. In particular, the combination of V/V-GP/P induced the highest number of total GC B cells followed by V/N/P and VP/NP/P regimens (*p* < 0.001). When we analyzed the percentage of HIV-1 gp140(96ZM651)-specific GC B cells (Figure 9A, right panel), we observed that the combination of VP/VP/P induced the highest HIV-1 Env-specific GC B cell activation followed by the combinations V/N/P and VP/NP/P (*p* < 0.001).

#### HIV-1 Env-Specific Tfh Cell Immune Response

CD4 T follicular helper (Tfh) cells are necessary for the development and maintenance of GC reactions, a crucial event that results in the generation of durable high affinity antibodies. This interplay between B and Tfh cells is mediated by different soluble and cell-associated factors, including CD40L(CD154), IL-10, IL-21, ICOS, and IL-4 (40). Since the frequency and quality of HIV-1-specific Tfh cells have been previously correlated with the generation of bNAbs (41, 42), we next decided to characterize by flow cytometry this specific cellular subset induced by the different immunization regimens at 10 days post-protein boost after the splenocytes were non-stimulated (RPMI) or stimulated *ex vivo* for 6 h with both HIV-1 gp140(96ZM651) peptide pools and protein. The gating strategy used for the identification of Tfh cells was the following: lymphocytes were gated on singlets followed by selection of live cells. CD4^+^CD8^−^ cells were then gated and analyzed based on the expression of CXCR5 and PD-1 markers. The double positive CXCR5^+^PD-1^+^ population defined total Tfh cells (Supplementary Figure 1B).

As shown in Figure 9B (left panel), the i.m. administration of the different combinations of VSV-GP-gp145, DNA-gp145, and NYVAC-gp145 vectors induced high levels of total CD4 T cells with Tfh phenotype (CXCR5^+^PD1^+^) in spleen compared with the 1.9% of total Tfh cells detected in naïve mice, although some differences were detected between the different groups. In particular, the percentages of total Tfh cells were higher in VSV-GP-gp145-primed animals compared to DNA-gp145-primed groups (*p* < 0.001) except for DP/DP/P (CI) group. Next, we evaluated the HIV-1 Env-specific Tfh cells by quantifying the percentage of CD4 Tfh cells that expressed CD40L and/or produced IL-4 and/or IFN-γ after Env peptide pool and protein stimulation in comparison with unstimulated cells (RPMI). As shown in Figure 9B (right panel), the combination of VSV-GP-gp145/NYVAC-gp145/P without the gp140 protein component during the first two immunizations (V/N/P) induced the highest HIV-1 Env-specific Tfh cell activation in spleen (*p* < 0.001).

#### HIV-1 Env-Specific CD4 and CD8 T Cell Immune Responses

Finally, we evaluated the HIV-1 gp140-specific CD4 and CD8 T cell immune responses induced by the different combinations of VSV-GP-gp145, DNA-gp145, and NYVAC-gp145 vectors. Splenocytes from immunized animals were stimulated *ex vivo* for 6 h with HIV-1 gp140(96ZM651) peptide pools and, after stimulation, cells were incubated with specific antibodies to identify T cell lineage (CD3, CD4, and CD8), effector cytokines (IL-2, IFN-γ, and TNF) and degranulation (CD107a) to define responding cells. The HIV-1 gp140-specific CD4 or CD8 T cell responses were established by the percentage of T cells with CD4 or CD8 phenotype that expressed CD107a and/or produced IL-2 and/or IFN-γ and/or TNF.

As shown in Figure 9C, HIV-1 gp140-specific T cell responses were polarized toward the CD4 compartment in DNA-gp145-primed groups and in the group VSV-GP-gp145/NYVAC-gp145/P. In contrast, in the other VSV-GP-gp145-primed groups and the protein only group, the HIV-1 gp140-specific T cells were distributed between CD4 and CD8 compartments. Regarding the magnitude of the HIV-1 gp140-specific response, the combination of VSV-GP-gp145/NYVAC-gp145/P induced the highest HIV-1 Env-specific CD4 and CD8 T cell responses (V/N/P) followed by the same combination but with the gp140 protein component co-administered with virus immunizations (VP/NP/P). In all immunization groups, the HIV-1 gp140-specific T cell responses were mostly of TEM phenotype (data not shown).

Overall, the results of this study in mice demonstrated that the combination of VSV-GP in the prime and NYVAC in the boost without protein component (V/N/P) triggered the highest HIV-1 Env-specific CD4, CD8, and Tfh cell immune responses with high levels of HIV-1 Env-specific GC B cells and binding antibodies followed by VP/NP/P regimen.

## Discussion

In spite of significant efforts in the design of immunization strategies able to achieve protective immune responses against HIV-1, there is yet no effective vaccine protocol to control virus spread. Multiple prime/boost strategies involving different classes of vectors and HIV-1 protein combinations to induce broad and multifunctional T and B cell immune responses have been tested in preclinical and clinical trials (9, 12). In this investigation, we showed that the combination of VSV-GP-gp145 in the prime and NYVAC-gp145 in the boost (V/N) triggered the highest and most balanced cellular and humoral responses to HIV-1 Env. When the vectors were delivered in reverse order or when prime/boost was performed with homologous vectors, independently of VSV-GP or NYVAC, the immune response was lower. Moreover, when purified HIV-1 gp140 protein (P) was administered as a late protein boost (V/N/P) or concurrently with the viral vectors during prime/boost immunizations and as a late protein boost (VP/NP/P), the highest Env-specific CD4, CD8 and Tfh cells were again observed in the V/N/P group showing that the effect of the protein component in the induction of HIV-1-specific T cell responses was more potent when it was only administered as a late protein boost than when it was co-administered with the viral vectors. Furthermore, this heterologous VSV-GP-gp145/NYVAC-gp145 combination was superior compared to DNA-primed regimens, regardless whether VSV-GP-gp145 or NYVAC-gp145 were included as booster components.

The 96ZM651 gp140 and 97CN54 gp140 sequences are both HIV-1 clade C originated from a South African isolate (96ZM651) and from a Chinese isolate (97CN54) and, therefore, are phylogenetically related but not identical (79% of protein sequence similarity). During immunizations, the different vectors (VSV, NYVAC, and DNA) encoded membrane-associated 96ZM651 gp145 envelope, whereas the purified gp140 protein used as protein component co-administered with the different vectors at prime and boost and as a late protein boost is derived from 97CN54 isolate. DNA and NYVAC vectors expressing 96ZM651 gp140 have been extensively tested in mice (19) and in NHPs (15, 43) and also used in a recent prophylactic phase 1b clinical trial (18). For these vectors, Env sequences were selected from 96ZM651 isolate with regard to future efficacy testing in high incidence regions such as in South Africa. Recruitment for a phase 2b efficacy trial (PrepVac) in South Africa is scheduled to start Jan 2020 using the Env-encoding DNA vaccine described in this manuscript together with the GMP grade 97CN54 gp140 protein, which was also used in this study. Therefore, for consistency reasons and also to allow a side-by-side comparison with the existing NYVAC and DNA vectors, we decided to generate a VSV vector encoding 96ZM651 gp145 protein. For the protein component, we consequently decided to use 97CN54 gp140 protein, which is available in GMP quality (one of the few HIV-1 Env trimeric proteins) and has already qualified for (and used in) clinical trials.

The major advantage of the immunization protocols used in the present work is the different nature of the virus-host interaction triggered during infection. VSV-GP is a replication-competent, highly attenuated RNA virus (21) and NYVAC is a replication-restricted, highly attenuated DNA virus (44). Both viruses efficiently expressed HIV-1 gp145 protein and are cytocidal, with VSV-GP-gp145 inducing a more rapid phosphorylation of the initiation factor eIF2α that regulates protein synthesis than NYVAC-gp145, correlating with apoptosis being more severe for VSV-GP-gp145 infection (Figure 2D). Since these studies were performed with a replication-competent (VSV-GP) and a replication-incompetent (NYVAC) viruses, it is unclear the immunogenic impact of the potential use of a replication-competent NYVAC-based vector in these prime/boost immunization protocols.

The HIV-1 clade C gp145 protein tested here comprises a 2nd generation construction (membrane-bound but with inactivated cleavage-site) and was designed to form trimers at the cell surface in order to trigger more potent antibody responses to Env than when the protein is released from cells. The trimeric structure of the HIV-1 gp145(96ZM651) protein expressed by the different vectors was confirmed by flow cytometry analysis of the binding profile of bNAbs to the membrane-bound HIV-1 gp145 protein expressed at the cell membrane of NYVAC-gp145-infected HeLa cells (Figure 3). This analysis indicates that the HIV-1 gp145 protein is expressed adopting a trimeric conformation since the panel of bNAbs used recognizes specific epitopes on the Env trimer. Further confirmation was obtained when this analysis was performed in HeLa cells infected with NYVAC recombinant viruses expressing a trimeric form of the HIV-1 Env protein or a monomeric form, where there was no recognition by bNAbs when the monomeric Env form was expressed, but instead a strong binding for the trimeric form was detected (data not shown). In addition, the punctate pattern observed by confocal microscopy in non-permeabilized HeLa cells infected with either VSV-GP-gp145 or NYVAC-gp145 viruses confirms the presence of HIV-1 gp145 protein at the cell membrane in a conformation that is clearly recognized by the human 10-1074 bNAb.

In the present work, we have reported with viral vectors that the prime/boost immunization regimen VSV-GP-gp145/NYVAC-gp145 (V/N) induced the highest binding antibody titers to Env, particularly during the adaptive phase. Similar binding antibody levels were observed in a different *in vivo* assay with the same immunization regimen and with gp140 protein component included [co-administered with the viral vectors and as a late protein boost (VP/NP/P) or only as a late protein boost (V/N/P)]. Since the different vectors encoded 96ZM651 Env, whereas the Env protein component co-administered with the vectors at prime and boost and as a late protein boost was of 97CN54 origin, both proteins were used for the serological analysis to look into both homologous and heterologous binding antibody responses. Due to the 79% protein sequence similarity between both proteins, no differences in reactivity were observed (Figure 7). The group VSV-GP-gp145/NYVAC-gp145 with only the gp140 protein component as a late protein boost (V/N/P) exhibited a heavily biased HIV-1 Env-specific response toward the Th1-associated IgG2a and IgG3 subclasses, while the other groups receiving the gp140 protein co-administered with the viral vectors exhibited Th2-associated IgG1 subclass responses. When DNA-gp145 was used as a priming component, neither VSV-GP-gp145 nor NYVAC-gp145 enhanced the binding antibody production against Env over the titers observed with the VSV-GP-gp145/NYVAC-gp145 combination.

Virus-specific bNAbs comprise the pivotal immune correlates of protection for the majority of successful viral-based vaccines. For HIV-1 infection, bNAbs have been reported to target different regions of the Env trimer. These regions include the variable loops 1 and 2 (V1/V2) glycans, the V3 glycan and the CD4 binding site on the gp120 protein, and the gp120/gp41 interface (45). Vaccination regimens able to elicit bNAbs with the capacity to neutralize the majority of HIV-1 strains are a main objective of the vaccine development field (46). In this work, a bNAb-binding assay was performed in HeLa cells infected with either VSV-GP-gp145 or NYVAC-gp145 vectors, showing that the HIV-1 gp145 protein expressed by both viruses is targeted to the cell membrane where it is recognized by a wide panel of bNAbs. This binding reactivity was more favorable for the HIV-1 gp145 protein expressed from NYVAC that from VSV-GP backbone; this could be most likely due to the more severe cytopathic effect attributed to VSV-GP, to the membrane alterations that occurred during the earlier induction of apoptosis in VSV-GP-gp145-infected cells or to a lower number of Env trimers present on the cell membrane of VSV-GP-gp145-infected HeLa cells. The reactivity to F105 non-NAb, the absence or low recognition level of bNAbs targeting V1/V2 quaternary epitopes together with the inactivation of the cleavage site in HIV-1 gp145 protein indicates a flexible and open close to native rather than a native-like trimer conformation adopted by the HIV-1 gp145 protein and that some non-native-like disordered Env trimers could also be produced during virus infection. In fact, this also occurs in infectious HIV virions *per se*, where functional Env trimer spikes, Env trimers that have shed one gp120, non-functional conformationally rearranged Env proteins or gp41 stumps can be present on virion membrane and available to elicit both NAb and non-NAb responses (47). However, since NYVAC- and DNA-based vaccines expressing HIV-1 gp140(96ZM651) have been used in preclinical studies in mice (19) and NHPs (15, 43) as well as in a phase 1b clinical trial (18) as mentioned above, the 96ZM651 gp145 was considered a useful model antigen to generate a recombinant VSV-GP vector expressing the same Env to compare the immunogenicity elicited by different vector combinations.

Despite the potential limitations of the 96ZM651 derived Env used in this study with regard to forming “perfectly closed, native-like” trimer structure, the results presented here clearly demonstrates excellent immunogenicity against HIV-1 Env protein, in particular when VSV-GP was used for priming followed by a NYVAC boost.

Neither the used model Env, nor the animal model (BALB/c) were selected to assess the induction of bNAbs since the assessment of neutralizing activity from mouse sera is difficult to determine due to the high background detected in non-immune serum. However, experiments in rabbits have shown the induction of tier 1A neutralizing antibodies upon immunization of rabbits with different VSV-GP-trimeric Env recombinant viruses (14). Additionally, a preclinical trial is ongoing in NHPs immunized with a VSV-GP-based vector expressing the trimeric gp140:G Env (extracellular Env part fused to transmembrane domain and cytoplasmatic tail of VSV G), which has been engineered and selected due to its capability to form close to perfect native-like trimers when exposed on the cell and/or virus surface, and with a protein component to define the capacity of the immunization regimen to elicit bNAbs.

Another important achievement of this work is the induction of specific immune cells that have been correlated with the control of HIV-1 infection, particularly GC B and Tfh cells. Within germinal centers (GCs), antigen-activated B cells are subjected to active somatic hypermutation (SHM), broadening the immunoglobulin genes and producing plasma cells that secrete somatically mutated high-affinity antibodies and memory B cells. The generation of HIV-1 bNAbs requires this extensive SHM (48), and the interaction of GC B cells with Tfh cells is responsible for the antibody affinity maturation (49, 50). Hence, the production of bNAbs is controlled by Tfh cells. In this context, preclinical and clinical studies have shown that the frequency of Tfh and GC B cells correlated with the development of bNAbs during simian immunodeficiency virus/simian-human immunodeficiency virus (SIV/SHIV) and HIV-1 infections (51–53). In normal individuals, a subset of highly functional blood-circulating memory Tfh cells have been identified and correlated with the capacity to produce HIV-1-specific bNAbs in HIV-1-infected individuals (52). This observation indicates that this Tfh subpopulation might be the target of novel vaccine candidates with the aim to provide optimal B cell help and, therefore, high levels of SHM that ultimately result in the induction of bNAbs. It has also been reported that early maintenance of a specific subset of peripheral Tfh cells strongly correlated with the breadth of the NAb responses detected during chronic HIV-1 infection. This observation suggests that the preservation of this peripheral Tfh cells should be needed to maintain the initial B cell activation required for affinity maturation, SHM and bNAbs development (54). It has also been reported that HIV-1-infected individuals with plasma bNAbs showed higher frequencies of resting memory Tfh cells compared to HIV-1-infected individuals without bNAbs (55). In summary, all these observations reinforce the concept of generating new vaccine candidates able to induce improved GC responses and, consequently, HIV-1 bNAbs production (41). The ability of the immunization regimen VSV-GP-gp145/NYVAC-gp145 (particularly with the gp140 component only administered as a late protein boost in the regimen V/N/P) to elicit high HIV-1-specific GC B and Tfh cells adds more value to this regimen in potentiating cell responses that might correlate with protection.

Additionally, HIV-1-specific CD8 T cells are also important for the control of HIV-1 replication (56). The relevance of the HIV-1-specific CD8 T cell responses has been reinforced by preclinical studies (57–60). Furthermore, the observation that there is a decrease of viremia after the induction of virus-specific CD8 T cells during HIV-1 infection emphasizes that this subset is required for the initial control of infection (61). In this context, we have shown that the immunization regimen VSV-GP-gp145 followed by NYVAC-gp145 (V/N/P) is the most effective at activating HIV-1-specific CD4 and CD8 T cells.

Overall, in this investigation we have established an immunization regimen based on the rhabdovirus VSV-GP and the poxvirus NYVAC vectors expressing optimized HIV-1 gp145 protein. When combined in prime/boost, with VSV-GP as the priming vector and NYVAC as the booster component and preferably with Env protein only administered as a late protein boost (V/N/P) and not also co-administered with viral vectors (VP/NP/P), this regimen resulted in a potent vaccination approach able to induce HIV-1-specific CD4, CD8, Tfh, and GC B cells and antibodies. This more balanced T and B cell activation represents a promising outcome of this novel combined vaccination strategy for future clinical studies on HIV-1.

## Data Availability Statement

The datasets generated for this study are available on request to the corresponding author.

## Ethics Statement

The mouse studies were reviewed and approved by Ethical Committee of Animal Experimentation (CEEA) of Centro Nacional de Biotecnología (CNB-CSIC, Madrid, Spain) according to International EU Guidelines 2010/63/UE on protection of animals used for experimentation and other scientific purposes, Spanish National Royal Decree RD 53/2013 and Spanish National Law 32/2007 on animal welfare, exploitation, transport, and sacrifice (permit number PROEX 281/16).

## Author Contributions

BP, CG, RW, JK, and ME: conceptualization. BP, CG, JG-A, CS-C, SW, DL, BA, CS, and DP: methodology. CSS: data analysis. ME and BP: writing-original draft. BP, CG, JG, SW, SD, RW, JK, and ME: writing-review and editing. YL, GP, and ME: funding acquisition. SD and ME: resources and supervision.

### Conflict of Interest

The authors declare that the research was conducted in the absence of any commercial or financial relationships that could be construed as a potential conflict of interest.
