# Supplementary figures and images for: Heterologous Combination of VSV-GP and NYVAC Vectors Expressing HIV-1 Trimeric gp145 Env as Vaccination Strategy to Induce Balanced B and T Cell Immune Responses
Source: Front Immunol. 2019 Dec 18;10:2941. doi: 10.3389/fimmu.2019.02941 (PMC6930178; doi:10.3389/fimmu.2019.02941)

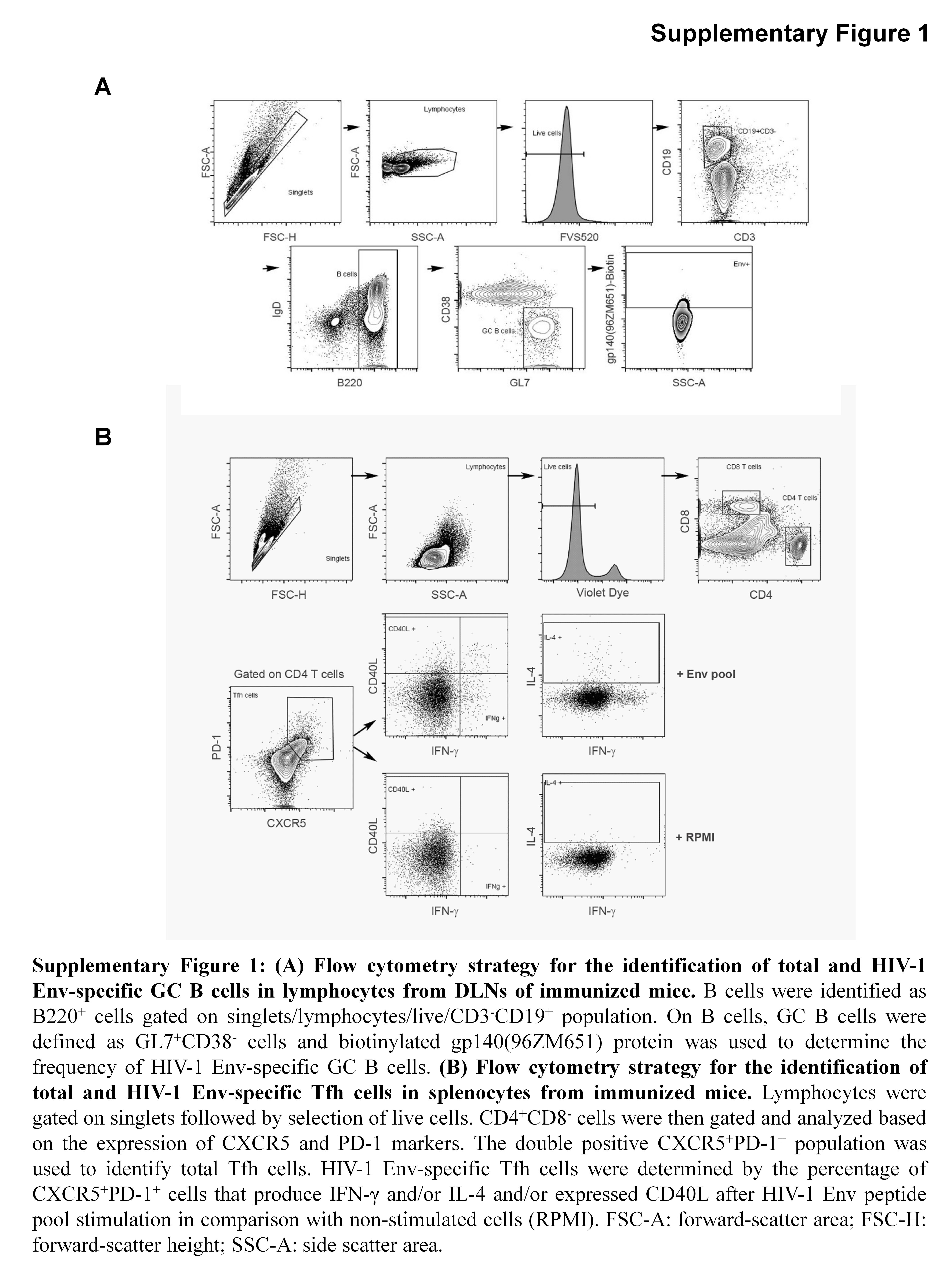

Supplement: Supplementary file 1 [file Image_1.jpg]
